# Inhibition of histone methyltransferase Smyd3 rescues NMDAR and cognitive deficits in a tauopathy mouse model

**DOI:** 10.1038/s41467-022-35749-6

**Published:** 2023-01-06

**Authors:** Jamal B. Williams, Qing Cao, Wei Wang, Young-Ho Lee, Luye Qin, Ping Zhong, Yong Ren, Kaijie Ma, Zhen Yan

**Affiliations:** Department of Physiology and Biophysics, State University of New York at Buffalo, Jacobs School of Medicine and Biomedical Sciences, Buffalo, NY 14203 USA

**Keywords:** Epigenetics and behaviour, Alzheimer's disease

## Abstract

Pleiotropic mechanisms have been implicated in Alzheimer’s disease (AD), including transcriptional dysregulation, protein misprocessing and synaptic dysfunction, but how they are mechanistically linked to induce cognitive deficits in AD is unclear. Here we find that the histone methyltransferase Smyd3, which catalyzes histone H3 lysine 4 trimethylation (H3K4me3) to activate gene transcription, is significantly elevated in prefrontal cortex (PFC) of AD patients and P301S Tau mice, a model of tauopathies. A short treatment with the Smyd3 inhibitor, BCI-121, rescues cognitive behavioral deficits, and restores synaptic NMDAR function and expression in PFC pyramidal neurons of P301S Tau mice. *Fbxo2*, which encodes an E3 ubiquitin ligase controlling the degradation of NMDAR subunits, is identified as a downstream target of Smyd3. Smyd3-induced upregulation of Fbxo2 in P301S Tau mice is linked to the increased NR1 ubiquitination. Fbxo2 knockdown in PFC leads to the recovery of NMDAR function and cognitive behaviors in P301S Tau mice. These data suggest an integrated mechanism and potential therapeutic strategy for AD.

## Abstract (web summary)

The study by Williams et al shows targeting the aberrant histone modifying enzyme Smyd3 rescues NMDAR and cognitive deficits in a mouse model of Alzheimer’s disease. It highlights the potential of epigenetic treatment in neurodegenerative diseases.

## Introduction

Neurodegenerative disorders are characterized by progressive synaptic/neuronal loss and are often accompanied by an uncontrollable aggregation of misfolded proteins^1,2^. In tauopathies, such as frontotemporal dementias (FTDs) and Alzheimer’s disease (AD), neurofibrillary tangles consisting of hyperphosphorylated tau^3,4^ are pathologic hallmarks closely linked to cognitive dysfunction^5–7^, however, the underlying mechanisms remain elusive.

Emerging evidence suggests that the onset and progression of AD is likely the result of a genetic predisposition in combination with environmental factors that lead to the dysregulation of key genes through epigenetic modifications^8–10^. Aging is the most obvious environmental risk factor for AD, however, AD represents only a subset of the aging population^11,12^. This suggests the possibility that those who develop AD may have an advanced form of aging, mediated by unique epigenetic modifications^9,13,14^. Defining the epigenetic mechanisms that underlie AD offers a promising strategy for gaining insights into the pathophysiological basis and treatment strategy for AD.

One of the major epigenetic mechanisms involved in transcriptional regulation is histone modifications. Aberrant histone acetylation has been implicated in AD^9,15,16^. Histone methylation, which can be linked to gene repression or activation, was also found to be altered in AD^16–19^. Particularly, we have found the upregulation of histone H3 methylation at lysine 4 (H3K4me3) in prefrontal cortex (PFC) of AD human postmortem tissues and P301S transgenic Tau mice^17^. In this study, we revealed the upregulation of a histone methyltransferase (HMT) Smyd3 (SET and MYND Domain Containing 3), which catalyzes the permissive H3K4me3, in AD. Interestingly, the upregulation of Smyd3-regulated H3K4me3 is associated with increased cell senescence, a hallmark of advanced aging^20^. The aberrant localization of H3K4me3 has also been found in early stage of AD^21^. We further identified *Fbxo2*, which encodes an E3 ubiquitin ligase controlling the degradation of NMDA receptor subunits^22,23^, as a key downstream target of Smyd3 that was dysregulated in a mouse model of tauopathies^24^. Inhibiting Smyd3 or knockdown of *Fbxo2* led to the rescue of synaptic and cognitive deficits in these mice. The findings have provided a multifaceted mechanism linking transcriptional dysregulation to synaptic dysfunction in tauopathies, and highlighted the therapeutic promise of epigenetic interventions.

## Results

### *Smyd3* is upregulated in PFC of AD patients, and Smyd3 inhibitor reverses the elevated H3K4me3 in a tauopathy mouse model

**Fig. 1:**
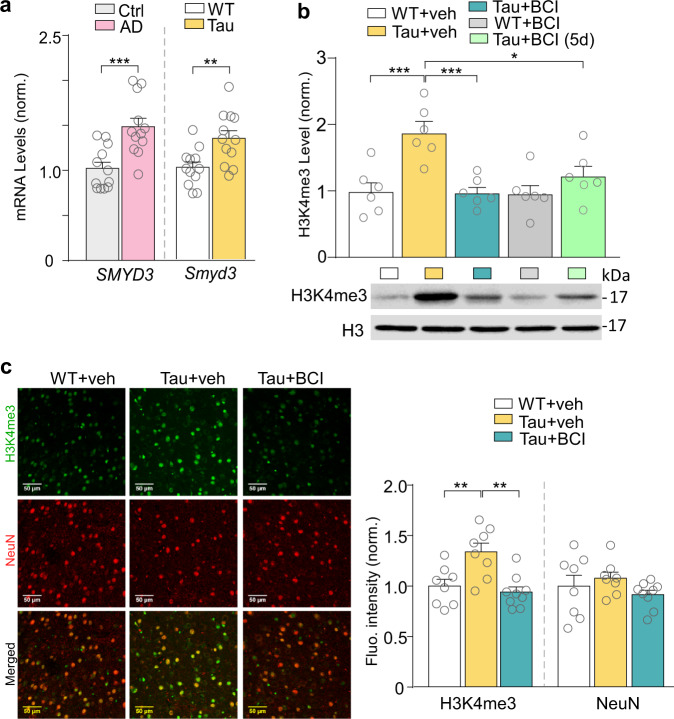
*Smyd3* is upregulated in AD, and treatment with Smyd3 inhibitor BCI-121 reverses the elevated H3K4me3 in PFC of P301S Tau mice. **a** Quantitative PCR analysis showing *SMYD3* mRNA levels in postmortem PFC from human control subjects and AD patients (*n* = 12 humans/group, *t* = 4.0, *p* = 0.0006, *t* test), and *Smyd3* mRNA levels in PFC of 6-month-old WT and Tau mice (*n* = 12 mice/group, *t* = 3.1, *p* = 0.005, *t* test). **b** Western blot data showing H3K4me3 protein levels in PFC of WT or Tau mice treated with BCI-121 (1 mg/kg, once daily for 3 days) or vehicle (*n* = 6 mice/group, *F*_4,25_ = 7.8, *p* < 0.0003, one-way ANOVA). All measurements were carried out at post-treatment day 1, except for Tau+BCI (5d), which was measured at 5 days after treatment. **c** Representative fluorescence images and quantitative analysis of H3K4me3 (green) and NeuN (red) fluorescence intensity in PFC slices from WT or Tau mice treated with BCI-121 or vehicle (*n* = 8–9 images/3 mice/group, H3K4me3, *F*_2,22_ = 9.7, *p* = 0.001, one-way ANOVA). In all figures, *p < 0.05, **p < 0.01, ***p < 0.001. Data are presented as mean values ± SEM. Detailed statistical data and full Western blots are included in Source Data files.

To find out epigenetic changes in AD, we first used postmortem PFC (Brodmann’s area 10) from AD patients (Braak stages V to VI). Detailed information on the human subjects is included in Supplementary Table 1. We found that the mRNA level of *Smyd3*, a gene encoding the H3K4me3 methyltransferase Smyd3 (KMT3E), was significantly upregulated in AD patients, compared to control subjects (Fig. 1a). Consistent with the human data, the mRNA level of *Smyd3* in PFC of P301S Tau transgenic mice (6-month old) was also significantly increased (Fig. 1a).

To determine whether inhibiting Smyd3 activity can normalize H3K4me3 level in Tau mice, we intraperitoneally (i.p.) administered the small molecule compound BCI-121, a specific Smyd3 inhibitor that competes against histones for Smyd3 binding, reduces H3K4 di- and trimethylation and downregulates Smyd3 target genes transcription^25,26^. Western blotting (Fig. 1b) revealed that Tau mice had significantly higher levels of H3K4me3, compared to WT mice, which was reversed by BCI-121 (1 mg/kg, once daily for 3 days) at post-treatment day 1. BCI-121 had little effect on H3K4me3 in WT mice, probably because of the low level of H3K4me3 in control conditions. Furthermore, at 5 days after BCL-121 treatment (3x) of Tau mice, H3K4me3 was still at the lower level, compared to untreated Tau mice, suggesting that this inhibitor has a long-lasting effect on histone methylation.

Immunostaining of H3K4me3 also confirmed that significantly elevated H3K4me3 fluorescence intensity in PFC neurons (NeuN-positive) of Tau mice was substantially reduced by BCI-121 treatment (Fig. 1c). Thus, Smyd3 is identified as a potential target to normalize H3K4me3 aberration in AD.

### Smyd3 inhibitor improves cognitive performance in a tauopathy model

**Fig. 2:**
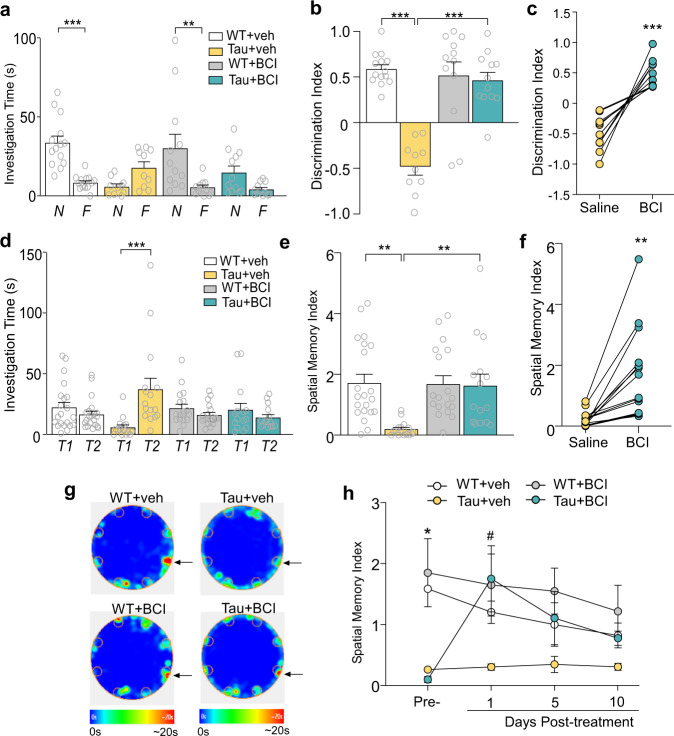
Treatment with Smyd3 inhibitor BCI-121 ameliorates cognitive deficits in P301S Tau mice. **a**, **b** Bar graphs showing investigation time on the novel (N) and familiar (F) objects (**a**), and discrimination indexes (**b**) for novel object recognition (NOR) tests of WT or Tau mice treated with BCI-121 or vehicle (*n* = 10–14 mice/group, **a**, F_3,88(interaction)_ = 8.4, *p* < 0.001; **b**, *F*_1,44(treatment)_ = 20.0, *p* < 0.0001; *F*_1,44(genotype)_ = 32.8, *p* < 0.0001, *F*_1,44(interaction)_ = 26.8, *p* < 0.0001, two-way ANOVA). **p < 0.01, ***p < 0.001. **c** Plot of NOR discrimination indexes in Tau mice pre- and post-treatment with BCI-121 or vehicle (*n* = 10 mice/group, *t* = 5.9, *p* = 0.0002, paired *t* test). ***p < 0.001. **d**, **e** Bar graphs showing investigation time on correct (T1) and incorrect (T2) holes (**d**), and spatial memory indexes (**e**) for Barnes maze (BM) tests of WT or Tau mice treated with BCI-121 or vehicl**e** (*n* = 15–21 mice/group, **d**, *F*_3,132(interaction)_ = 8.9, *p* < 0.0001; **e**, *F*_1,66(treatment)_ = 6.2, *p* = 0.02; *F*_1,66(genotype)_ = 8.0, *p* = 0.006, *F*_1,66(interaction)_ = 6.9, *p* = 0.01, two-way ANOVA). **p < 0.01, ***p < 0.001. **f** Plot of BM spatial memory indexes in Tau mice pre- and post-treatment with BCI-121 or vehicle (*n* = 15 mice/group, *t* = 4.2, *p* = 0.0008, paired *t* test). **p < 0.01. **g** Heatmaps of Barnes maze tests of different groups showing time at correct hole (arrow) vs incorrect holes. **h** Plot of BM spatial memory indexes of WT or Tau mice treated with BCI-121 or vehicle at different time points (*n* = 6 mice/group, *F*_3,20(interaction)_ = 11.0, *p* = 0.0002, two-way rmANOVA). *^, #^*p* < 0.05, *WT + veh vs. Tau+veh; ^#^Tau+veh vs. Tau+BCI. Data are presented as mean values ± SEM. Detailed statistical data are provided in Source Data files.

Next, we performed behavioral assays to examine the impact of Smyd3 inhibition on cognitive deficits in Tau mice. Novel object recognition (NOR) testing was first performed at 24 h after BCI-121 treatment (1 mg/kg, i.p., 3x). As shown in Fig. 2a, b, WT mice spent significantly more time on the novel object than the familiar object, while Tau mice lost the preference of the novel object, as indicated by a significantly lower discrimination index. However, Tau mice treated with BCI-121 displayed a markedly improved ability to distinguish the novel object from the familiar one, as shown by the restored discrimination index. Such improvement was observed in every individual Tau mouse we tested (Fig. 2c).

We then performed the Barnes maze (BM) spatial memory test, in which mice are trained to use visual cues to recall the location of a correct hole (8 holes in total) that had an escape box attached during the learning phases. As shown in Fig. 2d–g, during the memory phase, WT mice spent more time on the correct hole (escape box removed) than the 7 incorrect holes, while Tau mice spent significantly more time investigating the incorrect holes than the correct one, leading to a much lower spatial memory index. However, BCI-121-treated Tau mice spent more time investigating the correct hole, and displayed a significantly improved spatial memory index. To find out how long the therapeutic effect of Symd3 inhibition can last, we performed BM tests at 1, 5, and 10 days after BCI-121 treatment (1 mg/kg, 3x). We found that post-treatment day 1 had the largest difference in spatial memory index between BCI-121- vs. vehicle-treated Tau mice, however, BCI-121 administration still displayed moderate effects 5 and 10 days after treatment (Fig. 2h). Taken together, these behavioral data indicate that cognitive impairment in Tau mice can be effectively mitigated by inhibiting Smyd3.

### Smyd3 inhibitor ameliorates NMDAR deficits in a tauopathy model

**Fig. 3:**
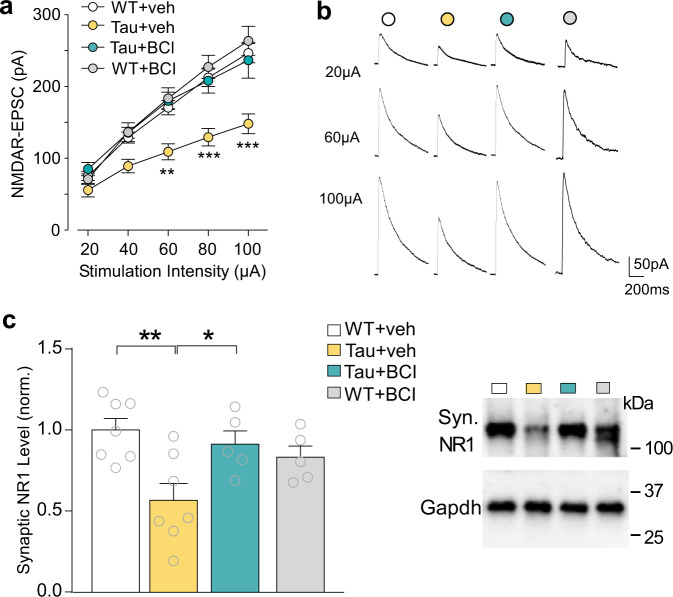
Treatment with Smyd3 inhibitor BCI-121 restores synaptic NMDAR function and expression in PFC of P301S Tau mice. **a** Input–output curves of NMDAR-EPSC in response to a series of stimulation intensities in PFC pyramidal neurons from WT or Tau mice treated with BCI-121 or vehicle (*n* = 12–26 cells/3–6 mice/group, *F*_3,62(group)_ = 8.0, *p* = 0.0001, two-way rmANOVA). **b** Representative NMDAR-EPSC traces. **c** Western blot data showing NR1 protein levels at the synaptic fraction in PFC of WT or Tau mice treated with BCI-121 or vehicle (*n* = 5–7 mice/group, *F*_1,20 (interaction)_ = 8.7, *p* = 0.008, two-way ANOVA). In all figures, *p < 0.05, **p < 0.01, ***p < 0.001. Data are presented as mean values ± SEM. Detailed statistical data and full Western blots are included in Source Data files.

Since PFC-mediated cognitive functions are highly dependent on glutamatergic signaling^27,28^, we next examined the impact of BCI-121 on synaptic function in PFC pyramidal neurons. We measured the electrical stimulation-evoked excitatory postsynaptic currents mediated by NMDA receptors (NMDAR-EPSC). As shown in Figs. 3a and 3b, input/output curves of NMDAR-EPSC evoked by various stimulation intensities were significantly diminished in Tau mice, consistent with our previous findings^17,19^, and BCI-121 treatment of Tau mice elevated NMDAR-EPSC to the control level. NMDAR-EPSC in WT mice was not significantly altered by BCI-121 treatment. These data highlight the ability of Smyd3 inhibition to restore the diminished NMDAR function in PFC pyramidal neurons of Tau mice.

To find out how BCL-121 inhibitor restores NMDAR function, we used Western blot analyses to examine the level of synaptic NMDARs. As shown in Fig. 3c, NR1 at the synaptic fraction of Tau mice was significantly reduced, compared to WT mice, which was significantly reversed by BCL-121 treatment, while synaptic NR1 in WT mice was not significantly altered by BCI-121 treatment. These data are consistent with the improvement of NMDAR function by Smyd3 inhibition in Tau mice.

### The E3 ubiquitin ligase Fbxo2 is identified as a key downstream target of Smyd3 in a tauopathy model

**Fig. 4:**
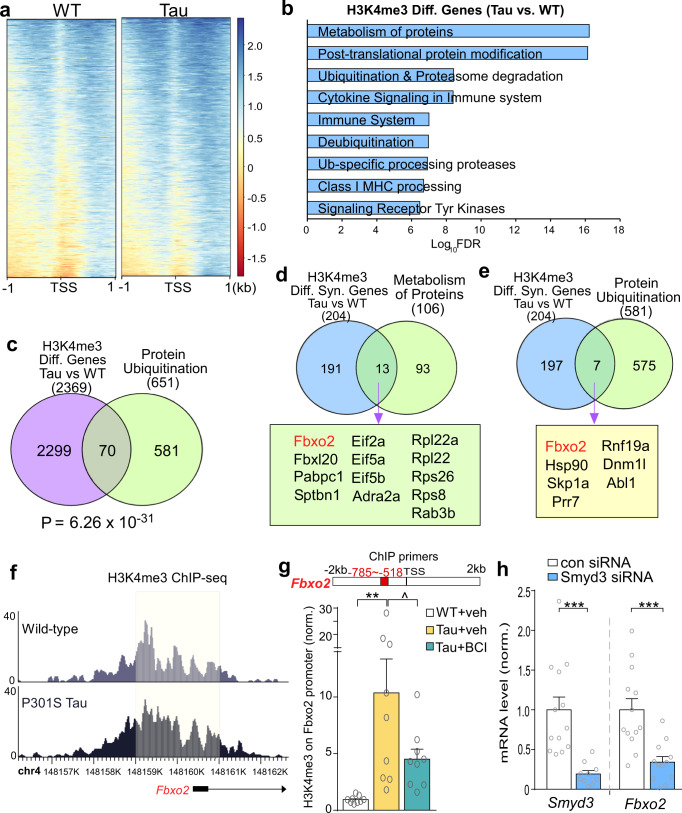
Genes with elevated H3K4me3 in Tau mice are enriched in protein ubiquitination pathway, and the E3 ubiquitin ligase Fbxo2 is identified as a key downstream target of Smyd3. **a** Heatmaps of H3K4me3 peaks within 1 kb of the TSS in WT and Tau mice. Scale bars: −2 (red) to 2.5 (blue). Note the difference on blue signals (darker blue for higher H3K4me3 enrichment). **b** Reactome pathway analysis showing the enriched categories of genes with increased H3K4me3 occupancy at their promoters in Tau mice, compared to WT mice. **c** Venn diagrams showing unique genes with increased H3K4me3 peaks in Tau mice significantly overlap with genes involved in protein ubiquitination (*p* = 6.26 × 10^−31^, one-sided hypergeometric *t* test). **d**, **e** Venn diagrams showing synaptic genes with increased H3K4me3 peaks in Tau mice overlap with genes involved in protein metabolism or protein ubiquitination. The overlapped gene lists are also shown. **f** ChIP-seq data showing the landscape of H3K4me3 peaks around TSS of *Fbxo2* gene in PFC from WT and Tau mice. The increased H3K4me3 binding sites in Tau mice are highlighted with a shadow box. **g** Bar graphs showing ChIP-PCR quantification of H3K4me3 enrichment at the promoter region of *Fbxo2* in PFC from WT or Tau mice treated with BCI-121 or vehicle (*n* = 9 mice/group, *F*_2,24_ = 6.96, *p* = 0.004, one-way ANOVA). **h** Quantitative PCR analysis showing *Smyd3 and Fbxo2* mRNA levels in N2A cells transfected with control siRNA vs. Smyd3 siRNA (*n* = 10–13 samples/3–4 cultures/group, *Smyd3*: *t* = 4.1, *p* = 0.0006, *Fbxo2*: *t* = 3.9, *p* = 0.0008, t-test). In all figures, ^*p* < 0.1, **p < 0.01, ***p < 0.001. Data are presented as mean values ± SEM. Detailed statistical data are provided in Source Data files.

To determine how upregulated Smyd3 is involved in cognitive and synaptic dysfunction in AD, we sought to investigate dysregulated downstream genes with elevated H3K4me3. To do so, we re-analyzed H3K4me3 ChIP-seq data between WT and Tau mice using genomic data (GSE179999) from our previous studies^17^ to detect differential peaks within 1 kb of the TSS region across the entire genome (Supplementary Fig. 1, Supplementary Data 1). Increased global H3K4me3 enrichment in Tau mice samples was observed (Fig. 4a). We then performed GO pathway analysis of genes with H3K4me3 peaks at their promoters that had a fold increase of at least 1.2 in Tau mice. We found that the most enriched pathways were associated with protein metabolism, post-translational modification and ubiquitination/degradation (Fig. 4b, Supplementary Data 2). These data highlight how permissive H3K4me3 can activate downstream mechanisms that disrupt normal protein homeostasis. Comparing genes with significantly altered H3K4me3 peaks at their promoters in Tau mice (2369) to genes involved in protein ubiquitination (651), 70 overlapped ones were identified (Fig. 4c), confirming the enrichment of protein ubiquitination genes with increased H3K4me3 in Tau mice.

Among the genes with increased H3K4me3 peaks at their promoters in Tau mice, 204 were identified as synaptic genes (Supplementary Data 3). Comparing these synaptic genes (204) with genes associated with protein metabolism (106), 13 overlapped ones were identified, including genes encoding F-box proteins involved in ubiquitination (*Fbxo2* and *Fbxl20*), translation initiation factors (*Eif2a*, *Eif5a*, and *Eif5b*), and ribosomal proteins (*Rpl22a*, *Rpl22*, *Rps26*, and *Rps8*) (Fig. 4d). Additionally, comparing the synaptic genes with increased H3K4me3 in Tau mice (204) and genes involved in protein ubiquitination (581), 7 overlapped ones were identified, including *Fbxo2*, which encodes the E3 ubiquitin ligase Fbxo2 (Fig. 4e). These data further suggest that the aberrant H3K4me3 in AD could diminish synaptic function via disrupted protein homeostasis.

ChIP-seq landscapes revealed a notable increase of H3K4me3 peaks at the *Fbxo2* promoter region in Tau mice, compared to WT mice (Fig. 4f). Our ChIP-PCR experiments (Fig. 4g) found that Tau mice displayed significantly higher levels of H3K4me3 occupancy at *Fbxo2* promoter than WT mice, while Tau mice treated with BCI-121 (1 mg/kg, 3x) exhibited a dramatic decrease of H3K4me3 occupancy, suggesting that the increased H3K4me3 enrichment at the *Fbxo2* promoter in Tau mice is driven by upregulated *Smyd3*.

To further support the direct role of Smyd3 in transcriptional regulation of *Fbxo2* gene, we performed qPCR experiments to examine the impact of Smyd3 knockdown on *Fbxo2* transcription. As shown in Fig. 4h, in the mouse cell line (N2A) transfected with Smyd3 siRNA, *Fbxo2* mRNA was significantly suppressed, suggesting that Smyd3 controls the activation of *Fbxo2* gene expression.

### Increased NMDAR ubiquitination in a tauopathy model is linked to Smyd3-induced Fbxo2 upregulation

**Fig. 5:**
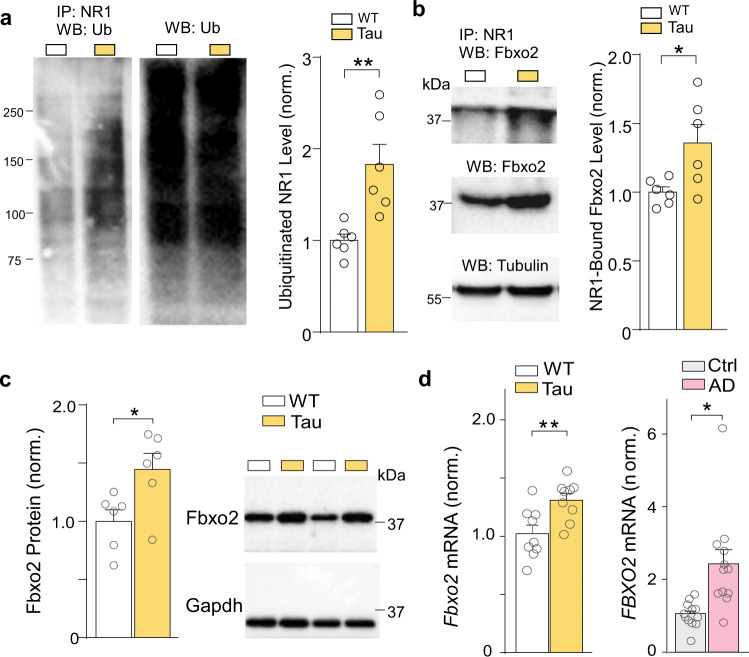
Increased NMDAR ubiquitination in Tau AD mice is linked to Fbxo2 upregulation. **a** Representative blots and quantification showing the ubiquitination of NR1 subunits in WT or Tau mice. Lysates of PFC slices were immunoprecipitated with anti-NR1, and then blotted with anti-ubiquitin (*n* = 6 mice/group, *t* = 3.6, *p* = 0.005, *t* test). Also shown are the immunoblots of ubiquitin as input controls. **b** Representative blots and quantification showing the Fbxo2-bound NR1 subunits in WT or Tau mice. Lysates of PFC slices were immunoprecipitated with anti-NR1, and then blotted with anti-Fbxo2 (*n* = 6 mice/group, *t* = 2.6, *p* = 0.03, *t* test). Also shown are the immunoblots of Fbxo2 and tubulin as loading controls. **c** Representative blots and quantification showing Fbxo2 expression in PFC of WT or Tau mice (*n* = 6 mice/group, *t* = 2.6, *p* = 0.02, *t* test). **d** Quantitative PCR analysis showing *Fbxo2* mRNA levels in PFC of WT and Tau mice (*n* = 9 mice/group, *t* = 3.1, *p* = 0.006, *t* test) and postmortem PFC from human control subjects and AD patients (*n* = 12 humans/group, *t* = 3.4, *p* = 0.003, *t* test). In all figures, *p < 0.05, **p < 0.01. Data are presented as mean values ± SEM. Detailed statistical data and full Western blots are included in Source Data files.

Fbxo2 is involved in the degradation of key synaptic proteins, such as the NMDA receptor subunits NR1, NR2A, and the postsynaptic marker PSD-95^22,23^. With *Fbxo2* identified as a key target gene dysregulated in Tau mice, we next examined NMDAR ubiquitination. Co-immunoprecipitation (Co-IP) analysis revealed a significant increase in the level of ubiquitinated NR1 in PFC of Tau mice, compared to WT (Fig. 5a). Co-IP of NR1 and Fbxo2 also revealed the increased level of Fbxo2-bound NR1 in Tau mice (Fig. 5b). Fbxo2 protein level in PFC, but not in hippocampus, was significantly increased in Tau mice, compared to WT (Fig. 5c, Supplementary Fig. 2a). In contrast, the E3 ligase responsible for AMPAR ubiquitination/degradation, Nedd4^23,29^, was largely unchanged in PFC and hippocampus of Tau mice (Supplementary Fig. 2b). Moreover, *Fbxo2* mRNA level was significantly increased in PFC of Tau mice and postmortem AD humans (Fig. 5d). These data suggest that *Fbxo2* is upregulated in PFC of AD due to the elevated H3K4me3 enrichment, leading to increased NR1 ubiquitination.

**Fig. 6:**
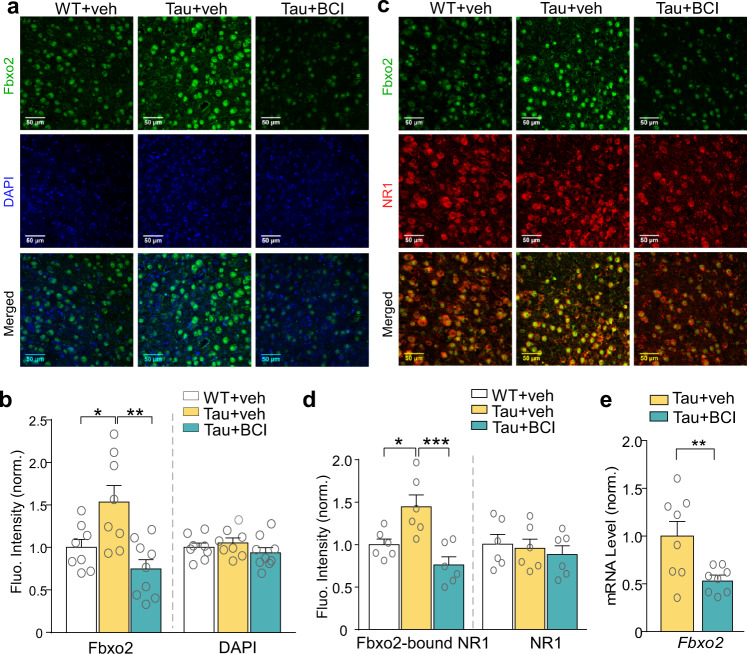
Treatment with Smyd3 inhibitor BCI-121 reverses the upregulated Fbxo2 and the elevated Fbxo2-bound NR1 in P301S Tau mice. **a**, **b** Representative fluorescence images (**a**) and quantitative analysis (**b**) of Fbxo2 (green) and DAPI (blue) fluorescence intensity in PFC slices from WT or Tau mice (6-month old) treated with BCI121 or vehicle (*n* = 8–9 images/3 mice/group, Fbxo2, *F*_2,22_ = 8.7, *p* = 0.002, one-way ANOVA). Scale bar: 50 μm. **c**, **d** Representative fluorescence images (**c**) and quantitative analysis (**d**) of Fbxo2 (green) and NR1 (red) fluorescence intensity in PFC slices from WT or Tau mice treated with BCI121 or vehicle (*n* = 6 images/2 mice/group, Fbxo2-bound NR1, *F*_2,15_ = 10.9, *p* = 0.0001, one-way ANOVA). Scale bar: 50 μm. **e** Quantitative PCR analysis showing mRNA levels of *Fbxo2* in PFC of Tau mice treated with BCI-121 or vehicle (*n* = 8 mice/group, *t* = 2.8, *p* = 0.02, *t* test). In all figures, *p < 0.05, **p < 0.01, ***p < 0.001. Data are presented as mean values ± SEM. Detailed statistical data are provided in Source Data files.

Next, we investigated whether Smyd3 is responsible for the elevated Fbxo2 expression in Tau mice. Quantitative immunostaining revealed the increased Fbxo2 fluorescence intensity in PFC neurons from Tau mice, compared to WT mice, which was significantly decreased by BCI-121 treatment (Fig. 6a and 6b). Furthermore, co-staining of Fbxo2 and NR1 (Fig. 6c, d) demonstrated that the fluorescence intensity of NR1 co-localized with Fbxo2 (Fbxo2-bound NR1) was significantly increased in PFC neurons from Tau mice, despite the unchanged total NR1 fluorescence intensity, and BCI-121 treatment of Tau mice brought down the level of Fbxo2-bound NR1 to the control level. In addition, qPCR experiments uncovered significantly decreased *Fbxo2* mRNA by BCI-121 treatment of Tau mice (Fig. 6e), further confirming the role of Smyd3 in regulating *Fbxo2* transcription.

### Knockdown of Fbxo2 in PFC of a tauopathy model restores NMDAR function and cognitive behaviors

**Fig. 7:**
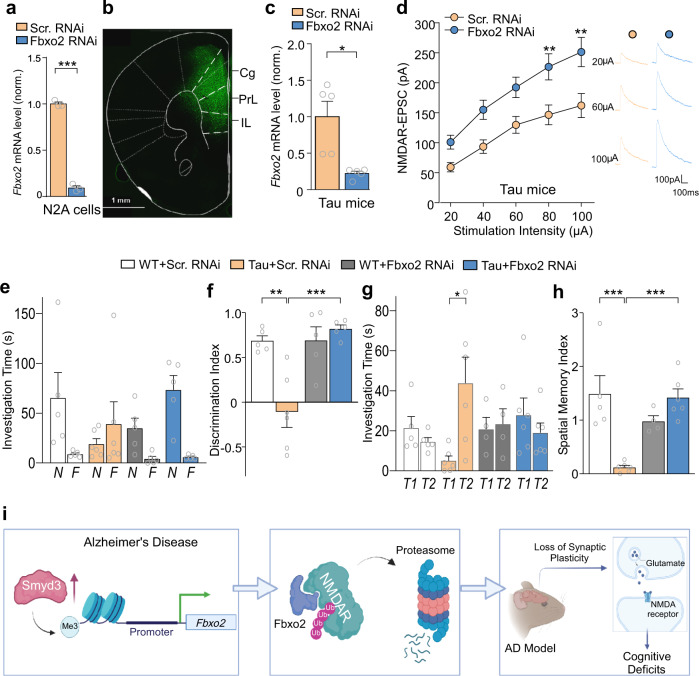
Knockdown of *Fbxo2* in PFC of P301S Tau mice restores NMDAR function and cognitive behaviors. **a** Quantitative PCR analysis showing mRNA levels of *Fbxo2* in N2A cells transfected with *Fbxo2* shRNA or a scrambled control shRNA (*n* = 3 cultures/group, *t* = 29.3, *p* < 0.0001, *t* test). **b** A representative fluorescent image showing viral-infected PFC from a mouse with the stereotaxic injection of *Fbxo2* shRNA AAV (GFP-tagged). Scale bar: 1000 μm. This experiment was repeated 3 times independently with similar results. **c** Quantitative PCR analysis showing *Fbxo2* mRNA levels in PFC of Tau mice infected with *Fbxo2* shRNA or scrambled shRNA AAV (n = 5 mice/group, *t* = 3.7, *p* = 0.02, *t* test). **d** Input–output curves of NMDAR-EPSC in response to a series of stimulation intensities in PFC layer V pyramidal neurons in Tau mice infected with *Fbxo2* shRNA or scrambled shRNA AAV (*n* = 13–16 cells/3 mice/group, *F*_1,27(group)_ = 9.1, *p* = 0.006, two-way rmANOVA). Inset: representative eEPSC traces at different stimulation intensities. **e**, **f** Bar graphs showing investigation time on novel (N) and familiar (F) objects (**e**), and discrimination indexes (**f**) for NOR tests of WT or Tau mice infected with *Fbxo2* shRNA or scrambled shRNA AAV (*n* = 4–6 mice/group, **e**, *F*_3,34(int**e**raction)_ = 4.1, *p* = 0.01; **f**, *F*_1,17(treatment)_ = 13.1, *p* = 0.002, *F*_1,17(interaction)_ = 12.9, *p* = 0.002, two-way ANOVA). **g**, **h** Bar graphs showing investigation time on correct (T1) and incorrect (T2) holes (**g**), and spatial memory indexes (**h**) for BM tests of WT or Tau mice infected with *Fbxo2* shRNA or scrambled shRNA AAV (*n* = 4–6 mice/group, **g**, *F*_3,34(interaction)_ = 4.9, *p* = 0.006; **h**, *F*_1,17(treatment)_ = 4.2, *p* = 0.06, *F*_1,17(interaction)_ = 21.8, *p* = 0.0002, two-way ANOVA). In all figures, *p < 0.05, **p < 0.01, ***p < 0.001. **i** A schematic model showing that Smyd3 elevation in AD leads to *Fbxo2* upregulation because of increased H3K4me3 occupancy, resulting in more NR1 ubiquitination and diminished NMDAR function in PFC, causing cognitive impairment. Smyd3 inhibition or Fbxo2 knockdown provides an intervention avenue to rescue synaptic and behavioral deficits in Tau AD mice. The diagram was created with Biorender. Data are presented as mean values ± SEM. Detailed statistical data are provided in Source Data files.

To find out whether upregulated *Fbxo2* contributes to synaptic and cognitive deficits in Tau mice, we generated a shRNA against Fbxo2 to knockdown *Fbxo2*, which was validated in an in vitro model (N2A cells, Fig. 7a). Then we injected an AAV containing the GFP-tagged *Fbxo2* shRNA into medial PFC of Tau mice (Fig. 7b). After 2–3 weeks of viral expression, a significant decrease of *Fbxo2* mRNA in vivo was confirmed via qPCR of infected PFC (Fig. 7c).

Electrophysiological recordings revealed that the I/O curves of NMDAR-EPSC were significantly elevated in PFC pyramidal neurons from Tau mice injected with *Fbxo2* shRNA AAV, compared to those injected with a scrambled control shRNA AAV (Fig. 7d). It suggests that NMDAR hypofunction is linked to upregulated Fbxo2 in Tau mice, which can be reversed by *Fbxo2* knockdown.

Finally, we investigated whether suppression of *Fbxo2* in PFC of Tau mice was effective in rescuing cognitive deficits. In NOR tests, compared to Tau mice injected with scrambled control shRNA AAV, Tau mice injected with *Fbxo2* shRNA AAV had a substantial increase of time spent on the novel object (Fig. 7e), and had significantly higher discrimination indexes (Fig. 7f). In BM spatial memory tests, Tau mice injected with *Fbxo2* shRNA AAV showed much more investigation time on the correct hole than Tau mice injected with scrambled control shRNA AAV (Fig. 7g), and had significantly improved spatial memory indexes (Fig. 7h). These data indicate that *Fbxo2* knockdown is capable of normalizing cognitive behaviors.

## Discussion

Multiple mechanisms have been implicated in AD, including maladaptive immune response^30–33^, mitochondrial dysfunction^34,35^, synaptic dysfunction^36–38^, and protein misprocessing^39–41^. How these pathways intersect is yet to be fully revealed. Recent genomic and epigenomic studies have identified the alteration of large networks of genes and their histone modifications in AD patients^9,13,16^ and AD-related mouse models^17–19^. We have demonstrated the feasibility of targeting different histone methylation enzymes in previous studies^17–19^, including identifying H3K4me3 as being aberrantly elevated due to the increased expression of MLL/SETD1 family histone methyltransferases (HMT) in P301S transgenic mice. Our work revealed that treatment of the P301S Tau mice with a small molecule compound WDR5-0103, which inhibits the catalytic activity of MLL/SETD1 by competing for their binding sites on the core subunit WDR5 in the complex, rescued the synaptic and behavioral deficits^17^. However, WDR5-0103 does not work on Smyd3, another HMT catalyzing H3K4me3. In addition, while we have explored the pathophysiological role of one of the gene targets that is upregulated by the elevated H3K4me3 in AD–Sgk1 (serum and glucocorticoid-regulated kinase 1)^17^, it is unclear how H3K4me3-mediated transcriptional dysregulation might be linked to pathways that are destructive to synaptic integrity in AD.

In this study, we have identified a novel mechanism that links several major disrupted biological processes in AD (Fig. 7i). We find that Smyd3 is elevated in PFC of AD, leading to the upregulation of genes involved in protein ubiquitination pathways, including the E3 ubiquitin ligase Fbxo2 that controls NMDAR ubiquitination/degradation. Consequently, NMDAR ubiquitination is increased and NMDAR-mediated synaptic function is diminished, leading to impaired cognitive behaviors. Inhibiting Smyd3 or knockdown of *Fbxo2* rescues synaptic and cognitive deficits in the mouse model of tauopathies.

Symd3 is a unique histone modifier containing both SET and MYND domains. Its function has largely been studied in cancer for tumorigenesis^42,43^. Smyd3 primarily catalyzes H3K4me2/3 and is also a component of the RNA polymerase complex^42^. Smyd3 upregulation in AD prompted us to target this epigenetic enzyme for intervention. Indeed, a short (3-day) treatment with the Smyd3 inhibitor BCI-121 ameliorates cognitive deficits in P301S Tau mice. Concomitantly, BCI-121 treatment significantly restores the diminished neuronal excitability and glutamatergic transmission in PFC of P301S Tau mice, providing a physiological basis for behavioral recovery.

In searching for downstream targets of Smyd3 that may be directly involved in synaptic dysfunction in AD, we find that genes with increased H3K4me3 at their promoters in Tau mice are most enriched in protein ubiquitination. Consistently, dysregulation of the ubiquitin proteasome system, a protein clearance mechanism, has been implicated in various neurodegenerative disorders^44–46^, including AD^39–41^. Protein folding and degradation is one of the top-ranking enriched pathways from proteomics studies of AD patients^47^. Here the E3 ubiquitin ligase *Fbxo2* is identified as one of the top synaptic genes involved in protein ubiquitination that have increased H3K4me3 occupancy in Tau mice. Consistently, transcriptomic studies have revealed *Fbxo2* as a gene associated with cognitive decline in AD^14,38^. The link of Smyd3 and *Fbxo2* is further demonstrated by the capability of Smyd3 inhibitor to reverse the elevated H3K4me3 occupancy at *Fbxo2* promoter and the elevated *Fbxo2* expression in Tau mice. Interestingly, the increased expression of *Symd3* and *Fbxo2* is also found in PFC excitatory neurons of a familial AD model expressing mutant APP/PS1 (5xFAD) from single-cell RNAseq data^48^.

Fbxo2 controls NMDAR subunit degradation^22,23^. The increased Fbxo2-bound NMDAR subunit NR1 in PFC of Tau mice is linked to the increased NR1 ubiquitination, which provides a potential mechanism for NMDAR hypofunction in AD. Viral-based knockdown of Fbxo2 in PFC restores NMDAR-EPSC, leading to the amelioration of cognitive deficits, further confirming the important role of Smyd3-Fbxo2-NR1 pathway in AD pathophysiology. Future studies will investigate the role of other downstream target genes of Smyd3 that may be involved in the regulation of PFC neuronal excitability, AMPAR-mediated synaptic transmission and other behaviors.

This study has linked epigenetic dysregulation of gene transcription to diminished expression of synaptic proteins in a model of tauopathy. There are several limitations to these findings. First, the contribution of the identified pathophysiological mechanisms to tau neuropathology and neurodegeneration is unclear. P301S Tau transgenic mice develop filamentous tau lesions at 6 months of age and neuron loss by 9–12 months of age^24^. We have uncovered the Smyd3-induced reduction of NMDARs in PFC of 6-month-old Tau mice that precedes the widespread neurodegeneration, suggesting that epigenetic aberrations may occur before overt synapse loss. Future studies are needed to examine temporal patterns of epigenetic changes in AD, the mechanisms underlying epigenetic aberrations in AD, and whether inhibiting Smyd3 to restore NMDARs can ameliorate tau hyperphosphorylation and slow down cognitive decline.

This study has focused on PFC, one of the key cognitive regions impaired in AD. P301S Tau transgenic mice exhibit hippocampal synapse loss and impaired synaptic function at 3 months of age before fibrillary tau tangles emerge^24^. Thus, another limitation is the complex brain circuits that might be involved in the rescuing effect of Smyd3 inhibitor on cognitive behaviors. PFC and hippocampus are interconnected, regulating memory processes in a collaborative manner. Although we found no observable changes in H3K4me3, Smyd3, or Fbxo2 in the hippocampus of Tau transgenic mice, we do not rule out that the behavioral changes could be related with synaptic loss in the hippocampus of Tau mice. Future studies are needed to examine spatial patterns of epigenetic changes in AD, and whether inhibiting Smyd3 in specific brain regions can improve certain aspects of behavioral abnormalities.

A third limitation is the unknown general applicability of the therapeutic strategy. In addition to tau pathology, Aβ accumulation is another major hallmark of AD. While our previous studies have found the elevated H3K4me3 in a familial AD model (5xFAD)^17^, it is unclear whether Smyd3-Fbxo2-NR1 pathway is also altered in 5xFAD mice. If so, future studies are needed to test whether inhibiting Smyd3 can ameliorate synaptic and cognitive deficits in 5xFAD mice.

While our results have provided a promising therapeutic strategy in a mouse model of AD, the translational value of this preclinical study awaits to be tested. There have been no clinical trials of SMYD3 inhibitors or other epigenetic compounds for AD treatment. Most of AD trials are on Aβ or p-tau deposits, which could be downstream consequences of some earlier pathophysiological triggers. The advantage of epigenetic treatment is the potential to normalize a network of genes in a sustained manner. The disadvantage is the potential off-target effects or side effects with prolonged applications. Before human trials, translational studies can be performed to test epigenetic abnormalities and interventions in human iPSC-derived neurons from AD patients.

## Methods

### Animals, Human Postmortem Tissues, and Compounds

Animal experiments were performed with the approval of the State University of New York at Buffalo Animal Care Committee (Protocol number: 202000049). Tau mice used were from the PS19 line, containing the T34 isoform of microtubule-associated protein tau with one N-terminal insert and four microtubule binding repeats (1N4R) encoding the human P301S mutation, which was expressed under the Prnp promoter^24^. These mice were obtained from Jackson Laboratories with the genetic background (C57BL/6 × C3H) F1, bred (noncarrier × hemizygote), and genotyped via PCR from tail DNA. P301S Tau mice and age-matched WT littermate controls were group-housed randomly (3-4 per cage) with ad libitum food accessibility in the 12-hr light-dark cycle (light: 6 am–6 pm; dark: 6 pm–6 am). Mice were kept at an ambient temperature of 23 °C and humidity of 51.4%. Both males and females (1:1 ratio) at the age of ~6 months old were used in the experiments. Animals were randomly assigned to different treatment groups.

Postmortem human frontal cortex (Brodmann’s area 10) from patients with AD and control subjects were provided by the National Institutes of Health (NIH) NeuroBioBank. NIH NeuroBioBank has followed all the ethical rules for the use of the human postmortem samples, and given us the permission of using them in our research. All individuals have given consent for their tissues to be used for research purposes. Upon arrival, tissue was stored in a −80 °C freezer until used for RNA isolation.

BCI-121 (Sigma-Aldrich) was immediately dissolved in dimethyl sulfoxide (DMSO) at a 20 mg/ml stock solution and stored at −20 °C. Prior to i.p. administration, BCI-121 was diluted in saline, and given at 1 mg/kg of body weight. Control mice were injected with an equivalent amount of vehicle (0.2% DMSO). All mice were injected with BCI-121 or vehicle once daily for three consecutive days and experiments were carried out at least 24 h after the final injection. Measurements were taken from distinct samples unless otherwise stated.

### Quantitative real-time PCR

Total RNA was isolated with the TRIzol reagent (Invitrogen), and the remaining DNA was removed by incubating with RNase-free DNase I (Invitrogen). Purified mRNA was then converted to cDNA with an iScript reverse transcription kit (Bio-Rad). Quantitative real-time PCR was performed on the iCycler iQ Real-Time PCR Detection System and iQ Supermix (Bio-Rad) according to the manufacturer’s instructions. Fold changes in the target genes were determined by: Fold change = 2^−Δ(ΔCt)^, where ΔCt = Ct(target)−Ct(GAPDH), and Δ(ΔCt) = ΔCt (AD)−ΔCt (control) or Δ(ΔCt) = ΔCt (Tau mice)−ΔCt (WT mice). Ct (threshold cycle) is defined as the fractional cycle number at which the fluorescence reaches 10x of the standard deviation of the baseline. A total reaction mixture of 20 μl was amplified in a 96-well thin-wall PCR plate (Bio-Rad) using the following PCR cycling parameters: 95 °C for 5 min followed by 40 cycles of 95 °C for 45 s, 55 °C for 45 s, and 72 °C for 45 s.

The following primers were used for qPCR: human *SMYD3* and mouse *Smyd3* (forward 5′-GAGAGGAGCTCACCATCTGC-3′; reverse 5′-CAGAACCTGCTCCCACTTCC-3′), human *FBXO2* (forward 5′-GTGTGGGGAAGAGGACTTGG-3′; reverse 5′-TCTTGACGCTCTCA TCGTGG-3′), mouse *Fbxo2* (forward, 5′-CGGAGACAATGGGGTGGAAT-3′; reverse, 5′-ACCCGAGTACCAGTCCTTCA-3′), human *GAPDH* (forward, 5′-GACAACAGCCTCAA GATCATCAG-3′; reverse, 5′-ATGGCATGGACTGTGGTCATGAG-3′), mouse *Gapdh* (forward, 5′-GACAACT CACTCAAGATTGTCAG-3′; reverse, 5′-ATGGCATGGACTGTGGTCATGAG-3′).

### Western blot

Nuclear extraction was performed as previously described^17^. In brief, micro-dissected PFC sections were homogenized in 1× hypotonic buffer [20 mM tris-HCl (pH 7.4), 10 mM NaCl, 3 mM MgCl_2_, 0.5% NP40, 1 mM PMSF, and cocktail protease inhibitor] and incubated on ice for 15 min. NP40 (10%) was added after the incubation and vortexed vigorously for 10 s. Then, homogenate was centrifuged at 3000 rpm for 10 min at 4 °C. The nuclear pellet was dissolved in cell extraction buffer [100 mM tris-HCl (pH 7.4), 100 mM NaCl, 1 mM EDTA, 1% Triton X-100, 0.1% SDS, 10% glycerol, and 1 mM PMSF, with cocktail protease inhibitor] for 30 min on ice with vortexing every 10 min. After a 30-min centrifugation at 14,000 × *g* at 4 °C, the supernatant was collected as the nuclear fraction.

Synaptosomal isolation was conducted as previously described^17^. PFC punches were collected from brain slices and homogenized in ice-cold lysis buffer with 10 mL/g (lysis buffer: 15 mM Tris, pH 7.6, 0.25 M sucrose, 1 mM EGTA, 2 mM EDTA, 25 mM NaF, 10 mM Na_4_P_2_O_7_, 10 mM Na_3_VO_4_). PMSF (1 mM) and a protease inhibitor tablet from Roche were added before use. A small portion of the lysate was collected as total protein extract. The remaining lysate was centrifuged at 800 × *g* for 10 min at 4 °C. Supernatant was transferred into a new tube and centrifuged at 10,000 × *g* for 10 min at 4 °C. The supernatant was collected as cytosolic fraction and the pellet was re-suspended in 1% Triton buffer with 300 mM NaCl, followed by a centrifugation at 16,000 × *g* for 15 min at 4 °C. Then the supernatant was collected as cytosolic proteins in synapses and the pellet was dissolved in 1% SDS buffer as membrane-associated proteins in synapses.

After getting the subcellular protein fraction, electrophoresis for protein samples were run on SDS gel at the appropriate size-selective percentage. Protein was then transferred to nitrocellulose membranes, and blocked in 5% milk (in TBS-T) for 1 h. Membranes were then incubated in a primary antibody at 4 °C overnight. Antibodies used include H3K4me3 (1:1000; Cell Signaling, 9751), H3 (1:1000; Cell Signaling, 4499), FBXO2 (1:500, Abcam, ab-28555), NEDD4 (1:1000; R&D Systems, MAB6218), NR1 (1:500, NeuroMab, 75-272) and GAPDH (1:2000; Cell Signaling, 5174). Membranes were then incubated in a mouse or rabbit HRP-linked secondary antibody (1:2000, GENXA931, GENA934, Millipore), and an ECL reaction was performed using enhanced chemiluminescence substrate (Thermo Fisher Scientific). Luminescence was detected by ChemiDoc XRS system (Bio-Rad), and density of blots was quantified by FIJI ImageJ software (v1.53u, NIH).

### Immunohistochemistry

Mice were anesthetized via isoflurane inhalation and transcardially perfused with PBS, followed by 4% paraformaldehyde (PFA) before brain extraction. Brains were post-fixed in 4% PFA overnight, then transferred to 30% sucrose in 0.1 M PBS solution for cryoprotection prior to slicing into 50 µm thick coronal sections. Slices were washed in PBS and blocked for 1 h in PBS containing 5% BSA and 0.05% Triton. After washing, slices were incubated with the primary antibody against H3K4me3 (1:1000; Abcam, ad8580), NeuN (1:200, Millipore, MAB377), Fbxo2 (1:100, Proteintech, 14590-1-AP), or NR1 (1:100, Neuromab, 75-272) overnight at 4 °C. After washing in PBS (15 min, three times), slices were incubated with secondary antibody Alexa Fluor 488 (1:1000; Thermo Fisher Scientific, A27034) or Alexa Fluor 594 (1:1000; Thermo Fisher Scientific, A-11032) for 1 h at room temperature (RT), followed by three washes with PBS. Slices were mounted on slides with VECTASHIELD mounting media (Vector Laboratories) with or without DAPI. Images were acquired using a Leica TCS SP8 confocal microscope and analyzed by FIJI ImageJ (v1.53u, NIH).

### Behavioral testing

All behavioral experiments were consistently conducted during the day (10:00 am–3:00 pm). Behavioral testing was performed following a 30-min period of acclimating to the behavioral room. All behavioral apparatus were cleaned with 70% ethanol and allowed to air dry prior to experimentation and between animal testing. Both genotype and treatment groups were blind to the experimenter during data acquisition for all behavioral experiments.

#### Barnes maze

Spatial memory was measured using Barnes maze as previously described^17,18^. Briefly, the mouse was placed on a round platform with eight equally spaced holes at the edge, one of which was attached with an escape box. The platform was surrounded by three spatial cues with a bright overhead light applied as a weak aversive stimulation to increase the motivation to escape from the circular platform. During the three learning phases (5-min interval) (information acquisition), the mouse was allowed to explore the platform using distal visual cues until finding the correct hole and entering the escape box. Once the mouse entered the escape box, it was fully covered and the mouse was transported back to its home cage between each training trial. Prior to the testing phase (memory recall), mice were placed in their home cage for 15 min. Testing was then conducted where the mouse was placed in the platform for 5 min with no escape box attached to the correct hole. The time spent on the correct hole (T1) and the other seven incorrect holes (T2) were measured. Spatial memory index was calculated by T1/T2.

#### Novel Object Recognition

The procedure consisted of three trials: habituation (no objects, 5 min), familiarization (two identical objects “familiar-A,” 5 min), and test phase [(familiar-A) and a new, different object (“novel-B”), 5 min] separated by a short delay period (5 min). The mouse was removed from the arena and placed in its holding cage in each interval between phases. All objects had similar size, textures, colors, but distinctive shapes. The objects were positioned counterbalanced. The arena and objects were cleaned between each trial with 70% alcohol to mask any olfactory cues. The room was illuminated by indirect white light. Exploration was defined by directing the nose at a distance of ≤2 cm to the object and/or touching it with the nose, while sitting on the object was not considered exploration. Total exploration time of the familiar and novel objects was recorded and used to calculate a discrimination index [time spent on novel object (B)—time spent on familiar object (A)]/[total time exploring both objects (B + A)] for test sessions.

All behavioral experiments were performed and analyzed without the knowledge of genotype and treatment. Heatmaps were generated using ANY-maze (ver. 6.0.3) (https://www.any-maze.com/).

### Electrophysiological recordings

All electrophysiological recordings were performed in layer V pyramidal neurons from coronal PFC slices (300 µm) as previously described^17,19^. Mice were anesthetized with 1-3% isoflurane (Sigma-Aldrich) prior to decapitation and brain extraction. The brain was then transferred to ice-cold sucrose solution where PFC sections were sliced on a VP1000S vibratome (Leica Microsystems Inc.). Slices were then submerged in oxygenated artificial cerebrospinal fluid [130 mM NaCl, 26 mM NaHCO_3_, 1 mM CaCl_2_, 5 mM MgCl_2_, 3 mM KCl, 1.25 mM NaH_2_PO_4_, and 10 mM glucose (pH 7.4); 300 mOsm]. PFC pyramidal neurons were visualized with a 40× water immersion lens and recorded with the MultiClamp 700 A amplifier (Molecular Devices, Sunnyvale, CA).

Evoked synaptic currents were generated with a pulse from a stimulation isolation unit controlled by a S48 pulse generator (Grass Technologies). A bipolar stimulating electrode (FHC, Bowdoinham, ME) was placed ~100 μm from the neuron under recording. For input-output responses, synaptic currents were elicited by a series of pulses with varying stimulation intensities (20 to 100 μA) delivered at 0.033 Hz. Patch electrodes contained the following internal solution: 130 mM Cs-methanesulfonate, 10 mM CsCl, 4 mM NaCl, 10 mM HEPES, 1 mM MgCl_2_, 5 mM EGTA, 2 mM QX-314, 12 mM phosphocreatine, 5 mM MgATP, 0.2 mM Na_3_GTP, and 0.1 mM leupeptin (pH 7.2–7.3; 265–270 mOsm). For recording NMDAR-EPSC, Bicuculline (20 μM) and CNQX (20 μM) were added to ACSF, and the cell (clamped at −70 mV) was depolarized to +40 mV for 3 s before stimulation to fully relieve the voltage-dependent Mg^2+^ block. Data analyses were performed with Clampfit (Axon instruments, Molecular Devices, Sunnyvale, CA) and GraphPad Prism 7 (GraphPad Software, Inc., La Jolla, CA).

### Genomic Data Analysis

H3K4me3 ChIP-seq data consisting of raw fastq files were obtained via *Gene Expression Omnibus* (GEO) under the accession number GSE179999. Reads were mapped to the mouse reference genome mm10 using Bowtie2 (v2.4.2)^49,50^ with default parameters. Mapped bam files were then filtered using samtools with a mapping quality (MAPQ) score cutoff of 20. Peak calling was then performed with MACS2 (v2.1.1.2 + )^51,52^ with a *p* < 0.05 cutoff for peak detection otherwise under default settings. Peaks were assessed using the default DESeq2 normalization method within DiffBind (v2.6.6.4.) R package^53^ for differential peak expression between Tau and WT samples. Peaks were then annotated with ChIPseeker (v1.8.0)^54^. To visualize genomic coverage, bigWig files were generated with bamCompare (v3.3.2.0.0) by deepTools^55^ with bin size set to 50 bp. Bigwig files were then visualized in the WashU Epigenome Browser (https://epigenomegateway.wustl.edu/) for further examination. These data were then analyzed with additional deepTools packages, in which bigWig files were prepared in computeMatrix (v3.1.2.0.0)^55^ for genomic regions within 1 kb of the TSS and plotted using plotHeatmap (v3.1.2.0.1)^55^.

Pathway analysis was performed using PANTHER (http://www.pantherdb.org/) on peaks within 1 kb of TSS and with a fold change greater than 1.5. An over-representation test was performed using Reactome pathway database against the Mus musculus reference genome. Significance was calculated using Fisher exact test and FDR correction. Synaptic genes were identified using SynGO analysis (https://www.syngoportal.org/index.html). A list of 204 synaptic genes from a *brain expressed* background gene set was aligned to H3K4me3 peaks in Tau mice and mapped to SynGO annotated terms.

### Chromatin Immunoprecipitation (ChIP)

Mouse cortex was dissected and homogenized in 250 μl ice-cold douncing buffer (10 mM Tris-HCl, 4 mM MgCl_2_,1 mM CaCl_2_, pH 7.5). The homogenized sample was incubated with 12.5 μl micrococcal nuclease (5 U/ml, Sigma, N5386) for 7 min and terminated by adding EDTA at a final concentration of 10 mM. Then hypotonic lysis buffer (1 ml) was added and incubated on ice for 1 hr. The supernatant was transferred to a new tube after centrifugation. After adding 10× incubation buffer (50 mM EDTA, 200 mM Tris-HCl, 500 mM NaCl), 10% of the supernatant was saved to serve as input control. To reduce nonspecific background, the supernatant was pre-cleared with 100 μl of salmon sperm DNA/protein A agarose-50% slurry (Millipore, 16–157) for 2 hr at 4 °C with agitation. The pre-cleared supernatant was incubated with an antibody against H3K4me3 (8 μg per reaction; ab8580, Abcam) overnight at 4 °C under constant rotation, followed by incubation with 60 μl of Salmon Sperm DNA/Protein A agarose-50% Slurry for 2 h at 4 °C. After washing for five times, bound complex was eluted twice from the beads by incubating with the elution buffer (100 μl) at room temperature. Proteins and RNA were removed by using proteinase K (Invitrogen) and RNase (Roche). Then immunoprecipitated DNA and input DNA were purified by QIAquick PCR purification Kit (Qiagen). Quantification of ChIP signals was calculated as percent input. Purified DNA was subjected to qPCR reactions with primers against mouse *Fbxo2* promoter (Forward, 5′-CTAGGCCCTCATGATCGCAG-3′; Reverse, 5′-AACGTAACCCGGCTAAGAGC-3′).

### N2A cell culture and siRNA transfection

Neuro-2a (N2a) cells were obtained from ATCC (CCL-131) and were routinely cultured in Eagle’s Minimum Essential Medium (EMEM, ATCC® 30-2003™) containing10% fetal bovine serum (Foundation, Gemini Bio-products), 1% penicillin/streptomycin (Invitrogen) and were maintained in a 5% CO_2_, 95% air humidified incubator at 37 °C. For routine culturing, cells were passaged once every 7 days, and medium renewal was performed 2 times per week.

SMYD3 siRNA (Santa Cruz biotechnology, sc-61576) and control siRNA (sc-37007) were prepared according to the manufacturer’s protocol. For each transfection, 80 pmol of siRNA was mixed with 100 μl of EMEM (ATCC® 30-2003™). 6 μl of siRNA transfection reagent (sc-29528) was mixed with 100 μl of EMEM. The two solutions were mixed and incubated for 30 min at room temperature. The cells were washed once with 2 ml of EMEM Medium, and the medium was removed. The transfection solution mixture was added to the washed cells, and the cells were incubated for 5 h at 37 °C in a CO_2_ incubator. 1 ml of EMEM + 10% FBS was added without removing the transfection mixture, and the cells were incubated for an additional 72 h before assay.

### Co-immunoprecipitation (Co-IP)

Frontal cortical slices from WT and Tau mice were collected and homogenized in lysis buffer (in mM: 50 NaCl, 30 sodium pyrophosphate, 50 NaF, 10 Tris, 5 EDTA, 0.1 Na_3_VO_4_, 1 PMSF, with 1% Triton X-100 and protease inhibitor tablet). Lysates were centrifuged (12,000 × *g*) at 4 °C for 15 min. Supernatant fraction was incubated with the antibody against NR1 (8 µl, NeuroMab, N308/48 2698) overnight at 4 °C, followed by incubation with 60 µl of protein A/G plus agarose (Santa Cruz Biotech., sc-2003) for 2 h at 4 °C. Immunoprecipitates were washed three times with lysis buffer, then boiled in 2 × SDS loading buffer for 5 min and separated on 7.5% SDS-polyacrylamide gels. Western blotting experiments were performed with anti-Ubiquitin (1:1,000, Cell Signaling, 43124), anti-Fbxo2 (1:1,000, Proteintech, 14590-1-AP) and anti-β-Tubulin (1: 5000, Sigma, T9026).

### Viral vector generation, validation and delivery

To knockdown *Fbxo2*, a short-hairpin RNA (shRNA) sequence (CCCAAGATGACAGCGTTAAGA) was generated and validated in the N2A cell line by qRT-PCR. The shRNA was cloned into GFP-tagged adeno-associated virus (AAV) vector (Addgene) under the control of U6 promotor. Viral particles (titer: 2.0 × 10^13^ vg/ml) were produced by the viral core center of Emory University. *Fbxo2* shRNA AAV was bilaterally injected to the medial PFC (2.0 mm anterior to bregma; 0.25 mm lateral; 2.0 mm deep; 0.5 μl each side) of mice as we described before^56^. In brief, mice were anesthetized with Ketamine & Xylazine (100 mg/kg and 5 mg/kg, i.p.) and placed on a stereotaxic apparatus (David Kopf Instruments, Tujunga, CA). The injection was carried out with a Hamilton syringe (needle gauge 31) at a speed of ~0.1 μl/min and the needle were kept in place for an additional 5 min. Two weeks after virus injection, animals were used for experiments.

### Statistical analysis

Data were analyzed with GraphPad Prism 7 (GraphPad), Clampfit (Molecular Devices, Sunnyvale, CA), and Mini analysis (Synaptosoft, NJ). All values are means ± SEM. All groups were tested for normality via Shapiro-Wilks tests. No sample was excluded from the analysis. The sample size was based on power analyses and was similar to those reported in previous works. Experiments were replicated in multiple cohorts of animals. The variance between groups being statistically compared was similar. Differences between two groups were assessed with unpaired two-tailed Student’s *t* test unless otherwise stated. Differences between more than two groups were assessed with one-way ANOVA, two-way ANOVA or two-way repeated measure ANOVA (rmANOVA), followed by *post hoc* Bonferroni tests for multiple comparisons.

### Reporting summary

Further information on research design is available in the Nature Portfolio Reporting Summary linked to this article.

## Supplementary information

Supplementary Information

Description of Additional Supplementary Files

Supplementary Data 1

Supplementary Data 2

Supplementary Data 3

Reporting Summary

## Data Availability

The ChIP-seq data generated previously^17^ and analyzed here have been deposited in the GEO public repository under accession code GSE179999, and can be accessed directly through the hyperlink. Both raw and processed data can be readily downloaded. Data processing and extracting methods are also included under the GEO accession code. Source data that contain all the raw data and statistics are provided with this paper. Source data are provided with this paper.

## Source data

Source Data
