# Supplementary material for: Inhibition of histone methyltransferase Smyd3 rescues NMDAR and cognitive deficits in a tauopathy mouse model
Source: Nat Commun. 2023 Jan 6;14:91. doi: 10.1038/s41467-022-35749-6 (PMC9822922; doi:10.1038/s41467-022-35749-6)
Supplement: Supplementary file 1 — Supplementary Information [file 41467_2022_35749_MOESM1_ESM.pdf]

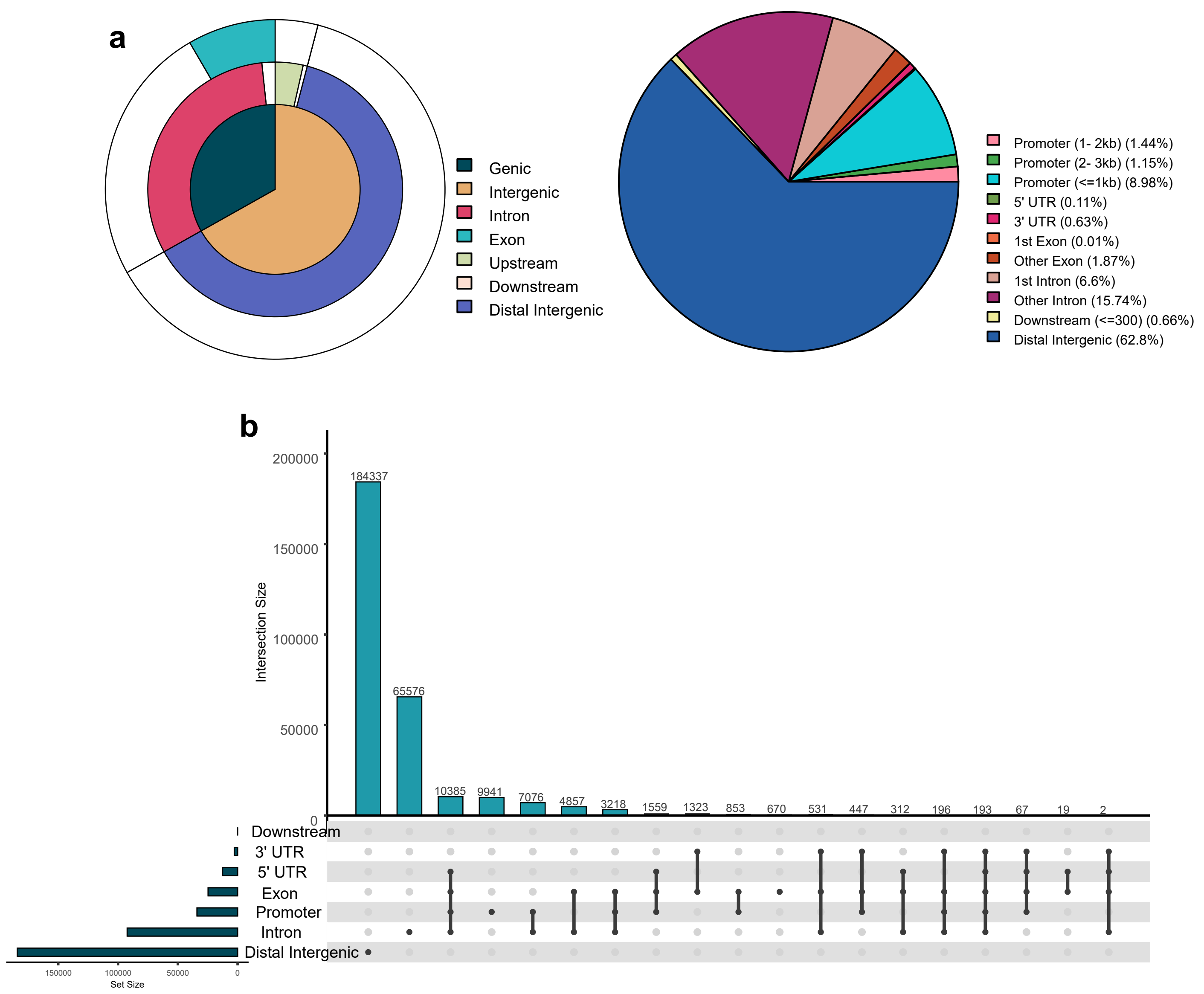

Supplementary Figure 1. Genomic features of differential expressed H3K4me3 peaks in WT and Tau mice from ChIP-seq data analysis.

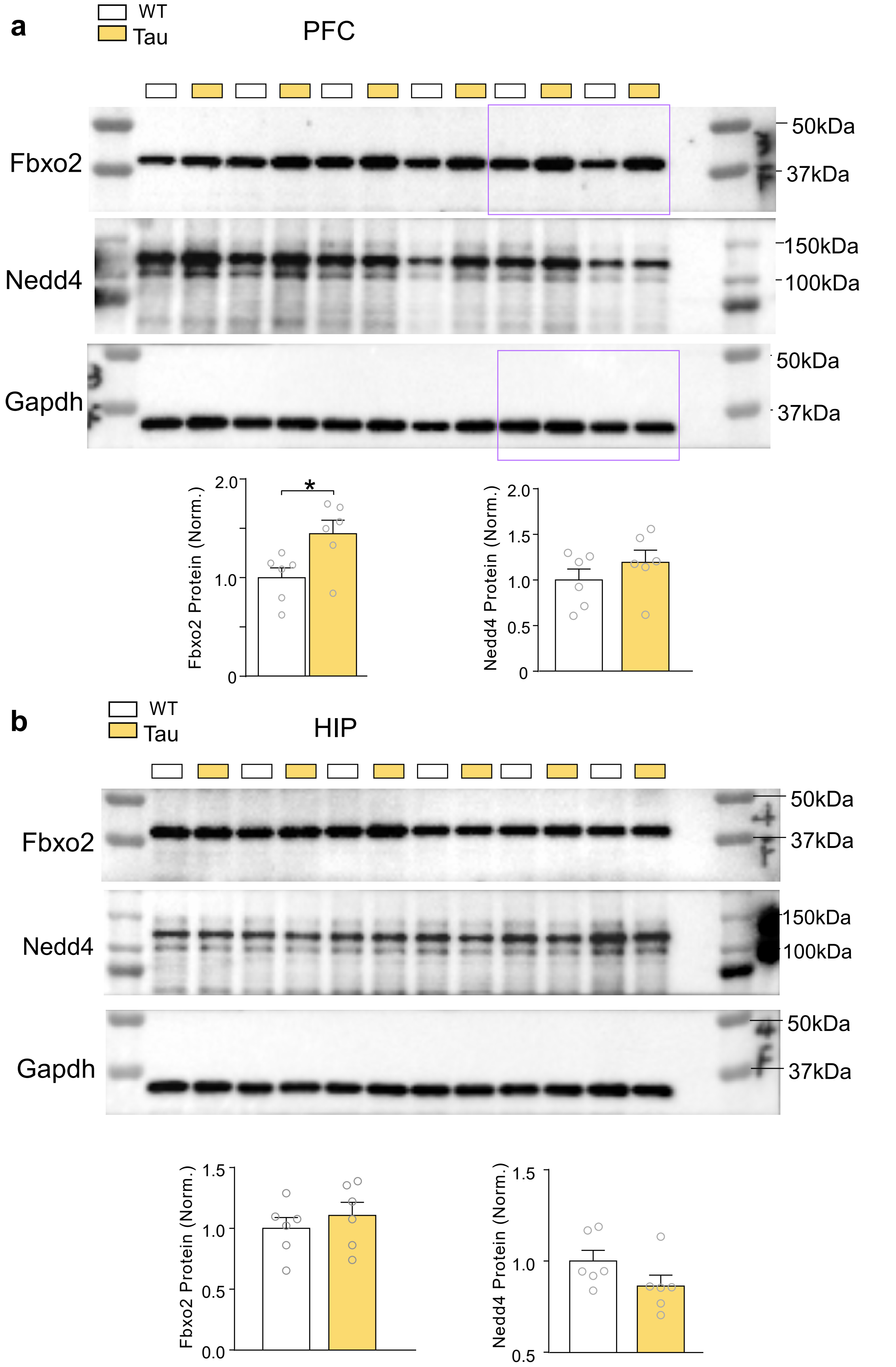

Supplementary Figure 2. Expression of Fbxo2 and Nedd4 in PFC and Hippocampus from WT and Tau mice (n=6 mice/group, a, Fbxo2: p=0.02, t-test). *: p<0.05. Data are presented as mean values ± SEM.

Sup. Table 1. Information on Human Postmortem Tissues

	Age (Years)	Sex	Neuropathology Diagnosis
#1	80+	Female	Alzheimer's
#2	60-70	Female	Alzheimer's
#3	70-80	Female	Alzheimer's
#4	70-80	Female	Alzheimer's
#5	70-80	Female	Alzheimer's
#6	80+	Female	Alzheimer's
#7	70-80	Female	Alzheimer's
#8	80+	Male	Alzheimer's
#9	70-80	Male	Alzheimer's
#10	80+	Male	Alzheimer's
#11	70-80	Male	Alzheimer's
#12	70-80	Male	Alzheimer's
#13	60-70	Female	Normal
#14	80+	Female	Normal
#15	70-80	Male	Normal
#16	80+	Male	Normal
#17	70-80	Male	Normal
#18	80+	Male	Normal
#19	60-70	Male	Normal
#20	80+	Male	Normal
#21	70-80	Male	Normal
#22	70-80	Female	Normal
#23	70-80	Male	Normal
#24	80+	Female	Normal
