# Supplementary material for: Inhibition of histone methyltransferase Smyd3 rescues NMDAR and cognitive deficits in a tauopathy mouse model
Source: Nat Commun. 2023 Jan 6;14:91. doi: 10.1038/s41467-022-35749-6 (PMC9822922; doi:10.1038/s41467-022-35749-6)
Supplement: Supplementary file 2 — Description of Additional Supplementary Files [file 41467_2022_35749_MOESM2_ESM.pdf]

Description of Additional Supplementary Files

File Name: Supplementary Data 1.

Description: Differentially Expressed H3K4me3 Peaks at Promoters in P301S Tau Mice. List of differentially expressed H3K4me3 peaks within ±1 kb TSS between Tau and WT, extracted from the public dataset GSE179999. MACS2 was used for peak calling with a threshold of $p<0.05$ and assessed for differential expression using DiffBind. P values were calculated individually at every base pair in the genome, then Benjamini-Hochberg was used to correct multiple comparisons or convert p values into q values or minimum FDR. Peaks with a fold change cutoff of 1.5 were then annotated with ChIPseeker.

File Name: Supplementary Data 2.

Description: Reactome Pathway Analysis of Differentially Expressed H3K4me3 Peaks at Promoters in P301S Tau Mice. Pathway analysis performed using PANTHER (http://www.pantherdb.org/) on H3K4me3 peaks within ±1 kb of TSS and with a fold change greater than 1.5. An over-representation test was performed using Reactome v65 against the Mus musculus reference genome. Significance was calculated using a two-tailed Fisher exact test and Benjamini-Hochberg correction for FDR. Raw p-value was determined by Fishers exact test or Binomial test, which is the probability based on null hypothesis that the number of genes in each category occurred by chance (randomly), as determined by the reference list.

File Name: Supplementary Data 3.

Description: Synaptic Genes with Increased H3K4me3 Occupancy at Promoters in P301S Tau Mice. List of 204 synaptic genes with differentially expressed H3K4me3 peak within ±1 kb of TSS in Tau mice mapped to SynGOannotated terms. For more information on SynGO analysis, visit https://www.syngoportal.org/index.html.
