# Supplementary material for: Inhibition of histone methyltransferase Smyd3 rescues NMDAR and cognitive deficits in a tauopathy mouse model
Source: Nat Commun. 2023 Jan 6;14:91. doi: 10.1038/s41467-022-35749-6 (PMC9822922; doi:10.1038/s41467-022-35749-6)
Supplement: Supplementary file 6 — Reporting Summary [file 41467_2022_35749_MOESM6_ESM.pdf]

nature portfolio

Corresponding author(s): Zhen Yan

Last updated by author(s): Nov 18, 2022

Reporting Summary

Nature Portfolio wishes to improve the reproducibility of the work that we publish. This form provides structure for consistency and transparency in reporting. For further information on Nature Portfolio policies, see our Editorial Policies and the Editorial Policy Checklist.

Statistics

For all statistical analyses, confirm that the following items are present in the figure legend, table legend, main text, or Methods section.

n/a	Confirmed	
☐	☒	The exact sample size (n) for each experimental group/condition, given as a discrete number and unit of measurement
☐	☒	A statement on whether measurements were taken from distinct samples or whether the same sample was measured repeatedly
☐	☒	The statistical test(s) used AND whether they are one- or two-sided<br>*Only common tests should be described solely by name; describe more complex techniques in the Methods section.*
☐	☒	A description of all covariates tested
☐	☒	A description of any assumptions or corrections, such as tests of normality and adjustment for multiple comparisons
☐	☒	A full description of the statistical parameters including central tendency (e.g. means) or other basic estimates (e.g. regression coefficient) AND variation (e.g. standard deviation) or associated estimates of uncertainty (e.g. confidence intervals)
☐	☒	For null hypothesis testing, the test statistic (e.g. F, t, r) with confidence intervals, effect sizes, degrees of freedom and P value noted<br>*Give P values as exact values whenever suitable.*
☒	☐	For Bayesian analysis, information on the choice of priors and Markov chain Monte Carlo settings
☒	☐	For hierarchical and complex designs, identification of the appropriate level for tests and full reporting of outcomes
☒	☐	Estimates of effect sizes (e.g. Cohen's d, Pearson's r), indicating how they were calculated

Our web collection on statistics for biologists contains articles on many of the points above.

Software and code

Policy information about availability of computer code

Data collection	Behavioral data were acquired with a computer running Any-Maze (version 6.0.3) tracking software.<br>Electrophysiological data were collected by Clampex software (MDS Analytical Technologies, Sunnyvale, CA).<br>Images were acquired by a Leica TCS SP8 confocal microscope (Leica Microsystems).<br>Images and WB were analyzed in FIJI ImageJ (v1.53u)<br>Quantitative real time PCR data were collected by iCycler iQ™ RealTime PCR Detection System.

Data analysis	Data analyses were performed with Clampfit 9, Mini Analysis and GraphPad Prism 7. Experiments with two groups were analyzed statistically using two-tailed Student's t-tests. Experiments with more than two groups were subjected to one-way ANOVA, two-way ANOVA, or two-way repeated measure ANOVA (rmANOVA), followed by post hoc Bonferroni tests.<br><br>ChIP sequencing data analyses were performed using the following tools:<br>Reads were mapped to the mouse reference genome mm10 using Bowtie2 (v2.4.2)<br>Peak calling was performed with MACS2 (v2.1.1.2+)<br>Peaks were assessed using the default DESeq2 normalization method within DiffBind (v2.6.6.4.) R package<br>Peaks were then annotated with ChIPseeker (v1.8.0)<br><br>To visualize genomic coverage, bigWig files were generated with bamCompare (v3.3.2.0.0) by deepTools<br>bigWig files were prepared in computeMatrix (v3.1.2.0.0)<br>Genomic regions within 1 kb of TSS were plotted using plotHeatmap (v3.1.2.0.1)

For manuscripts utilizing custom algorithms or software that are central to the research but not yet described in published literature, software must be made available to editors and reviewers. We strongly encourage code deposition in a community repository (e.g. GitHub). See the Nature Portfolio guidelines for submitting code & software for further information.

Data

Policy information about availability of data

All manuscripts must include a data availability statement. This statement should provide the following information, where applicable:

- Accession codes, unique identifiers, or web links for publicly available datasets
- A description of any restrictions on data availability
- For clinical datasets or third party data, please ensure that the statement adheres to our policy

The ChIP-seq data generated by our previous study (PMID: 33298440) have been deposited in the GEO public repository under accession code GSE179999 (https://www.ncbi.nlm.nih.gov/geo/query/acc.cgi?acc=GSE179999), and can be accessed directly through the hyperlink. Both raw and processed data can be readily downloaded. Data processing and extracting methods are also included under the GEO accession code. Source data are provided with this paper.

Human research participants

Policy information about studies involving human research participants and Sex and Gender in Research.

Reporting on sex and gender	Postmortem brain tissues from both genders of humans with or without Alzheimer's diease were used in this study.
Population characteristics	The information of the humans from whom postmortem tissues were obtained is included in a supplementary table.
Recruitment	Postmortem human frontal cortex (Brodmann's area 10) from patients with AD and control subjects were provided by the National Institutes of Health (NIH) NeuroBioBank. All individuals have given consent for their tissues to be used for research purposes.
Ethics oversight	NIH NeuroBioBank has followed all the ethical rules for the use of the human postmortem samples, and given us the permission of using them in our research.

Note that full information on the approval of the study protocol must also be provided in the manuscript.

Field-specific reporting

Please select the one below that is the best fit for your research. If you are not sure, read the appropriate sections before making your selection.

☒ Life sciences ☐ Behavioural & social sciences ☐ Ecological, evolutionary & environmental sciences

For a reference copy of the document with all sections, see nature.com/documents/nr-reporting-summary-flat.pdf

Life sciences study design

All studies must disclose on these points even when the disclosure is negative.

Sample size	The sample size was based on power analyses and was similar to those reported in previous works.
Data exclusions	No data except outliers were excluded.
Replication	Experiments were replicated in multiple cohorts of animals.
Randomization	Animals were randomly assigned to different treatment groups. Data from male and female mice were pooled together. No sex-based analyses have been performed, because of the general applicability of the investigated mechanisms in both sexes.

Blinding	Both genotype and treatment groups were blind to the experimenter during data acquisition for all behavioral experiments.

Reporting for specific materials, systems and methods

We require information from authors about some types of materials, experimental systems and methods used in many studies. Here, indicate whether each material, system or method listed is relevant to your study. If you are not sure if a list item applies to your research, read the appropriate section before selecting a response.

Materials & experimental systems

n/a	Involved in the study
☐	☒ Antibodies
☐	☒ Eukaryotic cell lines
☒	☐ Palaeontology and archaeology
☐	☒ Animals and other organisms
☒	☐ Clinical data
☒	☐ Dual use research of concern

Methods

n/a	Involved in the study
☒	☐ ChIP-seq
☒	☐ Flow cytometry
☒	☐ MRI-based neuroimaging

Antibodies

Antibodies used	Western Blot: H3K4me3 (1:1000; Cell Signaling, 9751), H3 (1:1000; Cell Signaling, 4499), FBXO2 (1:500, Abcam, ab-28555), NEDD4 (1:1000; R&D Systems, MAB6218), NR1 (1:500, Neuromab, 75-272), GAPDH (1:2000; Cell Signaling, 5174), beta-Tubulin (1: 5000, Sigma, T9026), mouse IgG HRP-linked secondary antibody (1:2000, Millipore, GENXA931) or rabbit IgG HRP-linked secondary antibody (1:2000, Millipore, GENA934).<br><br>Immunohistochemistry: H3K4me3 (1:1000; Abcam, ad8580), NeuN (1:200, Millipore, MAB377), Fbxo2 (1:100, Proteintech, 14590-1-AP), or NR1 (1:100, Neuromab, 75-272), Alexa Fluor 488 (1:1000; Thermo Fisher Scientific, A27034) or Alexa Fluor 594 (1:1000; Thermo Fisher Scientific, A-11032).
Validation	All antibodies have been validated by vendors or by our current and previously published studies (PMID: 30668640; 33298440).

Eukaryotic cell lines

Policy information about cell lines and Sex and Gender in Research

Cell line source(s)	Neuro-2a (N2a) cells were obtained from ATCC (CCL-131)
Authentication	N2A cells were cultured according to manufacture's protocol:<br>cultured in Eagle's Minimum Essential Medium (EMEM, ATCC® 30-2003™) containing 10% fetal bovine serum (Foundation, Gemini Bio-products), 1% penicillin/streptomycin (Invitrogen) and were maintained in a 5% CO2, 95% air humidified incubator at 37°C
Mycoplasma contamination	Negative
Commonly misidentified lines<br>(See ICLAC register)	No commonly misidentified cell lines were used in the study.

Animals and other research organisms

Policy information about studies involving animals; ARRIVE guidelines recommended for reporting animal research, and Sex and Gender in Research

Laboratory animals	6-7 months old WT mice (C57BL/6 x C3H) and transgenic PS19 (P301S) Tau mice were used in this study. Mice were group-housed randomly (3-4 per cage) with ad libitum food accessibility in the 12-hr light-dark cycle (light: 6am-6pm; dark: 6pm-6am). Mice were kept at an ambient temperature of 23°C and humidity of 51.4%.
Wild animals	No wild animals were used in this study.
Reporting on sex	Both sexes (males and females at 1:1 ratio) were used in this study.
Field-collected samples	This study did not involve samples collected from the field.
Ethics oversight	All experiments were performed with the approval of the Institutional Animal Care and Use Committee (IACUC) of the State University of New York at Buffalo (Protocol number: 202000049).

Note that full information on the approval of the study protocol must also be provided in the manuscript.
