# Supplementary material for: Inhibition of histone methyltransferase Smyd3 rescues NMDAR and cognitive deficits in a tauopathy mouse model
Source: Nat Commun. 2023 Jan 6;14:91. doi: 10.1038/s41467-022-35749-6 (PMC9822922; doi:10.1038/s41467-022-35749-6)
Supplement: Supplementary file 7 — Source Data [file 41467_2022_35749_MOESM7_ESM.zip › Source Data-Full blots.pdf]

Full blots (Fig. 1b)

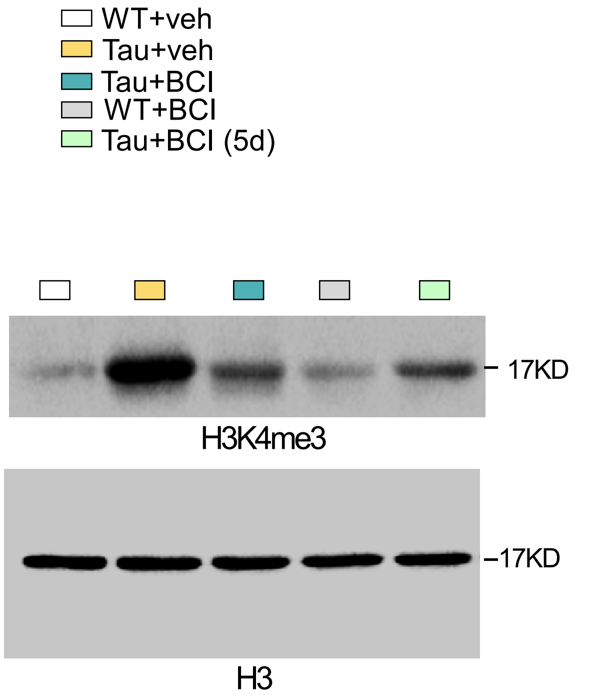

Full blots (Fig. 3c)

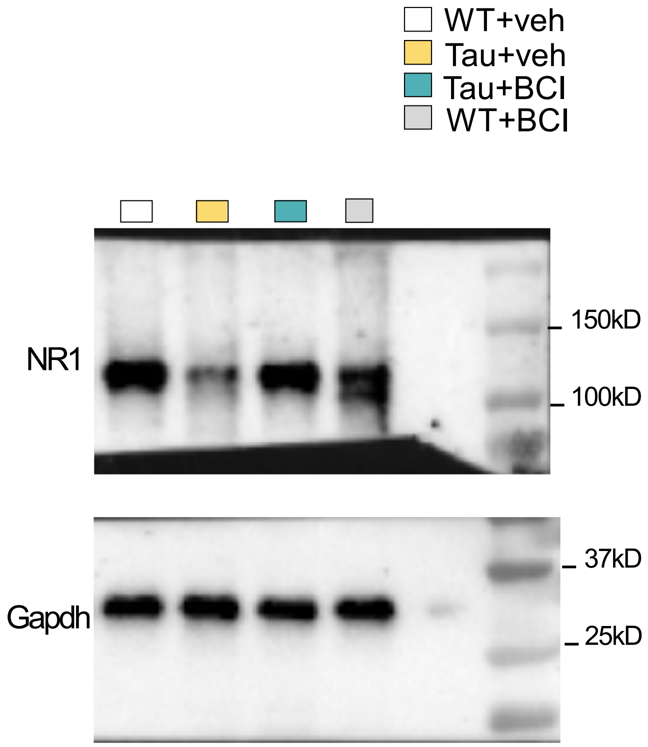

Full blots (Fig. 5b)

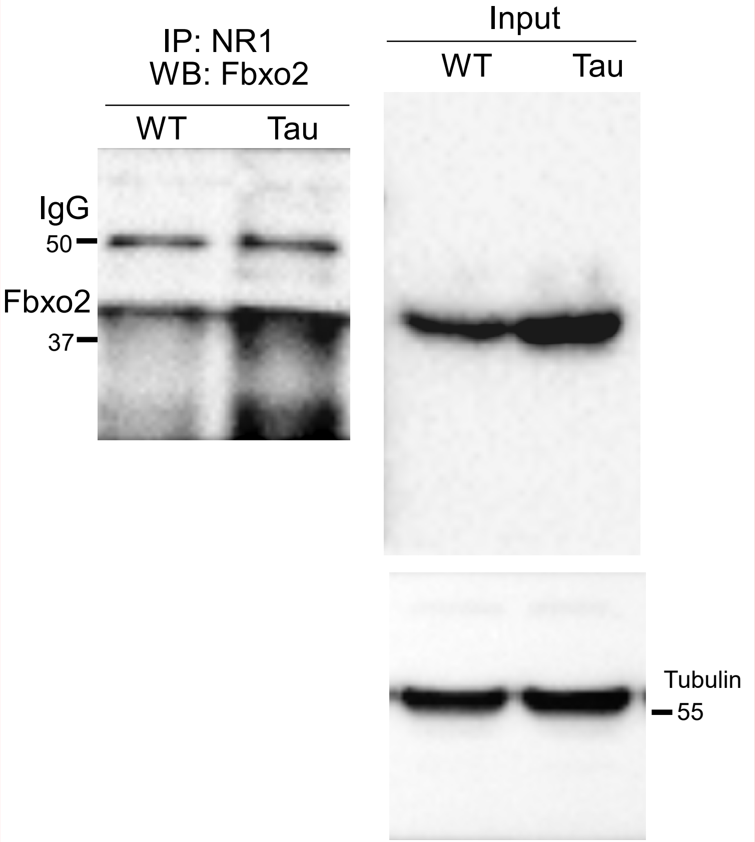

Full blots (Fig. 5c)

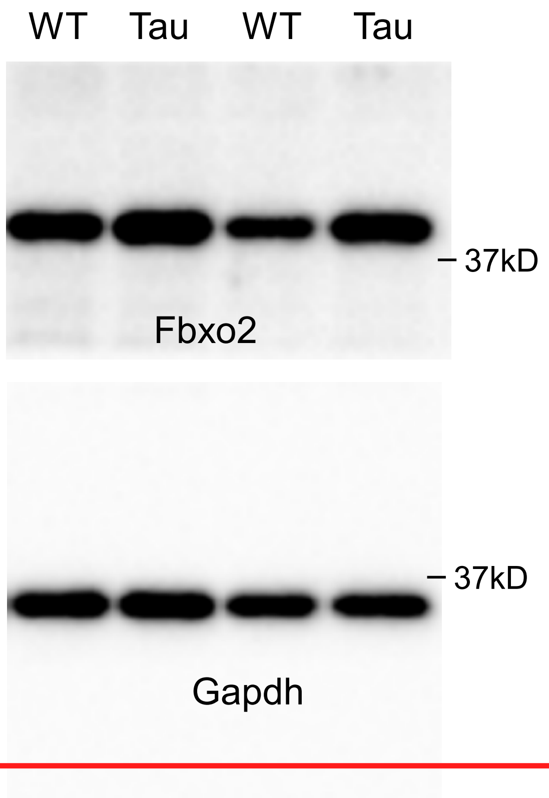

Source Data: Full Western blots. Related to Figures 1, 3, and 5.
